# Retrospection

**Published:** 1899-12

**Authors:** 


### RETROSPECTION.

THE natural tendency of the mind is to review the past, and to the average reader the history of human effort and its failures is more interesting than the present experiences or anticipations of the future; hence the thought at the beginning of the last month of the year turns naturally to retrospection. This is, or should be, equally the duty of professions and individuals. The business man takes an account of stock at this period, closes his books for the year, and the balance sheet will show loss or gain.

The profession that fails to do this year by year may make history, but it cannot properly appreciate it, and, in not appreciating it, it equally fails to note defects or to remedy them.

The first half of the present century found dentistry made up of widely separated and strongly individualized operators, and these possessed neither retrospective nor anticipatory qualities, or, at least, failed to manifest them. This was natural for the period, and in nowise reflects upon the intelligence of the workers of that day. The time in which we are active is altogether different, and the man who fails to keep in touch with the history of his profession, whether this be for a day, a week, a year, or through the centuries, is lacking in that which goes to make up the rather undefined term, professional spirit.

The century of work does not close for another year, notwithstanding some would have us believe that 1899 finishes the century, and it is, therefore, unnecessary to include the work of this period in the outlook of the past.

The question that concerns us now is not what was done by the fathers, but what we are doing, or have been doing the past twelve months, to make our profession worthy of our own respect and that of the world at large. This is, perhaps, a difficult question to answer. History cannot truthfully be written while the scenes it attempts to portray are still fresh in the mind of the observer. The broad view that the historian is supposed to take is not possible for the active participant. The soldier sees but a small part of the conflict in which he is engaged. This is equally true of each member of the professional army. All have a specified work to do, and are generally doing it well, but the sum total of effort and its influence for good or ill cannot be computed in a limited period. When Spooner introduced arsenic for the devitalization of pulps, neither he nor his contemporaries could possibly have anticipated its far-reaching influence on the dental mind. In their view it was simply a means to remove an obstruction, and that obstruction was a hyperæsthetic pulp; but as we look at it now, its introduction led to an entire change in methods, and from this, apparently, simple procedure arose more perfected processes in pulp-canal treatment, new forms in instruments, a higher appreciation of the value of teeth, and eventually led up to a determination to make the forceps the very last resort of the practitioner. The philosophical historian can, therefore, deliberate upon causes and see the results more effectually in proportion to the distance he is removed from the immediate activities. The year, therefore, has a limited outlook, but while its sphere of observation is a narrow one, it nevertheless is useful as a factor in mental growth.

The one now nearly past has not been remarkable from our limited visual range, and yet, when the activity of the members of the dental profession is considered, it must take rank with the best of the years preceding. While, with few exceptions, the papers presented in our various organizations have not been remarkable for original work, their average value has been worthy of note; in fact, more readable matter has been presented, both at home and abroad, than usual in the same period. This was especially noticeable at the National Dental Association, but dental readers must have observed the same marked improvement in all the leading dental organizations. While there have been but few papers presented based on original investigations, these have been of decided value and have added materially to our knowledge. The dental profession is fast growing out of its swaddling-clothes, and does not value the writer who says, "I think," or "I believe," but does honor the one who can prove his position; or, in other words, absolute knowledge has become a power, and mere verbiage, however panoplied in technicalities, is being relegated to deserved obscurity.

There has not been the past year that progress along the line of college work so manifest in former periods. Perhaps this is well, as too rapid growth leads to weakness. It is by no means certain that all that has been done in the direction of dental college training will eventually prove of value. We are too near the centre of activity to judge, but it would seem as though present tendencies will not prove advantageous to dental education. Without harmony progress is impossible, and there is too much contention over matters of minor importance to make the present outlook altogether hopeful. That the inharmonious will be settled in time there can be no question, but the antagonism engendered by State enactments was not quieted at Niagara, although an excellent beginning was made there which materially brightens the prospects for the future. Some progress has been made in interstate recognition, and it is hoped that ere long the incongruous condition of one State legislating against another will have ceased as a disturbing element.

There has been a marked improvement in our periodical literature. Those journals devoted to dentistry and issued by tradehouses are, apparently, making a decided effort to elevate the tone. The supply houses are beginning to recognize the importance of having men of broad intelligence at the helm, and that these must be untrammelled by trade influences. While independent dental journalism cannot flourish under commercial trammels, it is a satisfaction to note that progress is being made here, and this fact demonstrates very conclusively that the advance in dental training is beginning to have its influence and is permeating the mass of the so-called dental literature of the country as well as the minds of those who control it.

The books issued under dental supervision are growing in value, but the time has not yet arrived when a student can say, "In this volume is enclosed all that I need for my foundation work." When dentistry has a series of text-books in which everything is eliminated except that which bears directly and practically upon the subject in hand, such as Gray's "Anatomy," we may look for better-trained students. Several of this character have been issued, but more are needed. Our text-books are loaded down with matter valuable in itself, but wholly unsuited as *pabulum* for the untrained mind.

The past year has not added anything to our practical knowledge, but this could not have been expected. Future changes, if any, will be slowly evolved. They may come in a year, but more likely it will be many years before anything revolutionary in practice will be developed.

We can leave our retrospection of the past year with the comfortable feeling that if there has not been a marked advance, there has been no retrogression, and that the influence of the past twelve months is all in the direction of progress, and this augurs well for the position of dentistry in the coming century.

